# Realization of Opened and Closed Nodal Lines and Four- and Three-fold Degenerate Nodal Points in XPt (X = Sc, Y, La) Intermetallic Compound: A Computational Modeling Study

**DOI:** 10.3389/fchem.2020.609118

**Published:** 2020-11-05

**Authors:** Heju Xu

**Affiliations:** College of Science, North China University of Science and Technology, Tangshan, China

**Keywords:** 4-fold degenerate nodal point, triply degenerate nodal point (TNP), spin-orbit coupling (SOC), topological element, phonon dispersion

## Abstract

Realizing rich topological elements in topological materials has attracted increasing attention in the fields of chemistry, physics, and materials science. Topological semimetals/metals are classified into three main types: nodal-point, nodal-line, and nodal-surface types with zero-, one-, and two-dimensional topological elements, respectively. This study reports that XPt (X = Sc, Y, La) intermetallic compounds are topological metals with opened and closed nodal lines, and triply degenerate nodal points (TNPs) when the spin–orbit coupling (SOC) is ignored. Based on the calculated phonon dispersions, one can find that ScPt and YPt are dynamically stable whereas LaPt is not. When SOC is added, the one-dimensional nodal line and zero-dimensional TNPs disappear. Interestingly, a new zero-dimensional topological element, that is, Dirac points with 4-fold degenerate isolated band crossings with linear band dispersion appear. The proposed materials can be considered a good platform to realize zero- and one-dimensional topological elements in a single compound and to study the relationship between zero- and one-dimensional topological elements.

## Introduction

In the last decade, with the discovery of topological insulators (Cava et al., 2013; Kou et al., 2013; Zhao et al., 2013; Shen and Cha, 2014; Wang et al., 2014; Luo et al., 2015; Zhou et al., 2015; Liu et al., 2016; Chen et al., 2017a; Loïc and Izmaylov, 2017; Pan et al., 2017; Pielnhofer et al., 2017; Politano et al., 2017; Andrey et al., 2018; Hu et al., 2018, 2019; Gao et al., 2019; Mal et al., 2019; Qiao et al., 2019; Narimani et al., 2020), topologically non-trivial materials have attracted significant interest in the chemistry, physics, and materials science communities. Recently, studies have increasingly focused on topological semimetals/metals (Bin et al., 2018; Chenguang et al., 2018; Zhou et al., 2018; He et al., 2019, 2020; Jin et al., 2019a, 2020b; Li et al., 2019; Qie et al., 2019; Xie et al., 2019; Yi et al., 2019; Zhong et al., 2019; Ma and Sun, 2020; Meng et al., 2020b; Wang et al., 2020a,c,d; Yang and Zhang, 2020; Zhang et al., 2020; Zhao et al., 2020) with non-trivial band topology. For example, in 2018, Schoop et al. (2018) described the key features of the electronic structures of topological semimetals/metals and how these structures can be realized based on chemical principles.

Topological semimetals/metals can be roughly classified into three main parts: nodal-point (Chen et al., 2015; Yuan et al., 2017; Zhang et al., 2017a,c, 2018c; Jing and Heine, 2018; Ma et al., 2018; Tsipas et al., 2018; Khoury et al., 2019), nodal-line (Chang et al., 2016; Liu et al., 2018b; Guo et al., 2019; Sankar et al., 2019; Tang et al., 2019; Xu et al., 2019; Zhang et al., 2019; Jin et al., 2020a; Kirby et al., 2020; Zhou et al., 2020), and nodal-surface (Türker and Sergej, 2018; Wu et al., 2018; Zhang et al., 2018b,d; Fu et al., 2019; Yang et al., 2019b, 2020; Chen et al., 2020; Wang et al., 2020e; Xiao et al., 2020) semimetals/metals enjoying zero-, one-, and two-dimensional topological elements, respectively. The main examples of nodal-point semimetals/metals are Weyl and Dirac semimetals/metals with 2- and 4-fold degenerate band-crossing points with linear dispersion. In addition, 3-, 6-, and 8-fold (Cano and Vergniory, 2016; Lu et al., 2016; Weng et al., 2016b) band degenerates also exist. Among them, nodal-point semimetals/metals with 3-fold band degenerates [i.e., triply degenerate nodal point (TNP)] are of importance owing to their special properties. Many investigations have been conducted to predict and confirm new TNP semimetals/metals (Weng et al., 2016a; Xia and Li, 2017; Zhang et al., 2017b,d; Guo et al., 2018; Owerre, 2018; Jin et al., 2019b; Yang et al., 2019a). For example, in 2019, Jin et al. (2019b) reported that centrosymmetric Li_2_NaN is a topological material with critical-type TNPs. A critical-type TNP is an interesting topological metal phase that lies between type-I and type-II TNPs. In 2018, Guo et al. (2018) proposed that YRh_6_Ge_4_, LaRh_6_Ge_4_, and LuRh_6_Ge_4_ are TNP materials, and what is more, Zhu et al. (2020) performed transport measurements and confirmed TNP fermions in YRh_6_Ge_4_. In 2019, Yang et al. (2019a) experimentally demonstrated TNP as well as double Fermi arc surface states in a three-dimensional phononic crystal.

Nodal-line semimetals/metals with one-dimensional topological elements may show various forms according to the shape of the nodal lines, such as nodal link (Yan et al., 2017), nodal chain (Yan et al., 2018), nodal box (Sheng et al., 2017), nodal ring (Zhang et al., 2018a; Wang et al., 2020b), nodal knot (Bi et al., 2017; Lee et al., 2018), and nodal net (Feng et al., 2018). For example, in 2018, Zhou et al. (2018) proposed that two-dimensional B_2_C hosts opened and closed nodal-line states based on first-principles calculation. In 2020, Yi et al. (2019) predicted that NaAlGe and NaAlSi nodal-line materials would be good cathode materials for sodium ion batteries. In 2020, Wang et al. (2020d) proposed that a two-dimensional Nb_3_GeTe_6_ monolayer is a topological nodal-line material with a nearly flat nodal line around the Fermi level and that it led to a remarkable thermoelectric power factor platform. In 2018, Liu et al. (2018a) proposed that graphene monolith, a three-dimensional nodal-line semimetal, is a candidate lithium ion battery anode material. In 2019, Yan et al. (2019) proposed that the Cu_2_Si monolayer is a topological material with possible superconductivity and nodal-line fermions.

The electronic structure, dynamical stability, and topological signatures of ScPt, YPt, and LaPt, a cubic-type family of materials with $\text{Pm}\bar{3}\text{m}$ space group are investigated in the present study. This study shows that opened and closed nodal lines and 3-fold degenerate nodal-point states can be realized in ScPt, LaPt, and LuPt when the spin-orbit coupling is ignored. Moreover, the effect of spin-orbit coupling on the topological signatures of these systems is also considered. A 3- to 4-fold degenerate nodal-point transition can be found in these systems when spin-orbit coupling is added.

## Materials

**Figure 1:**
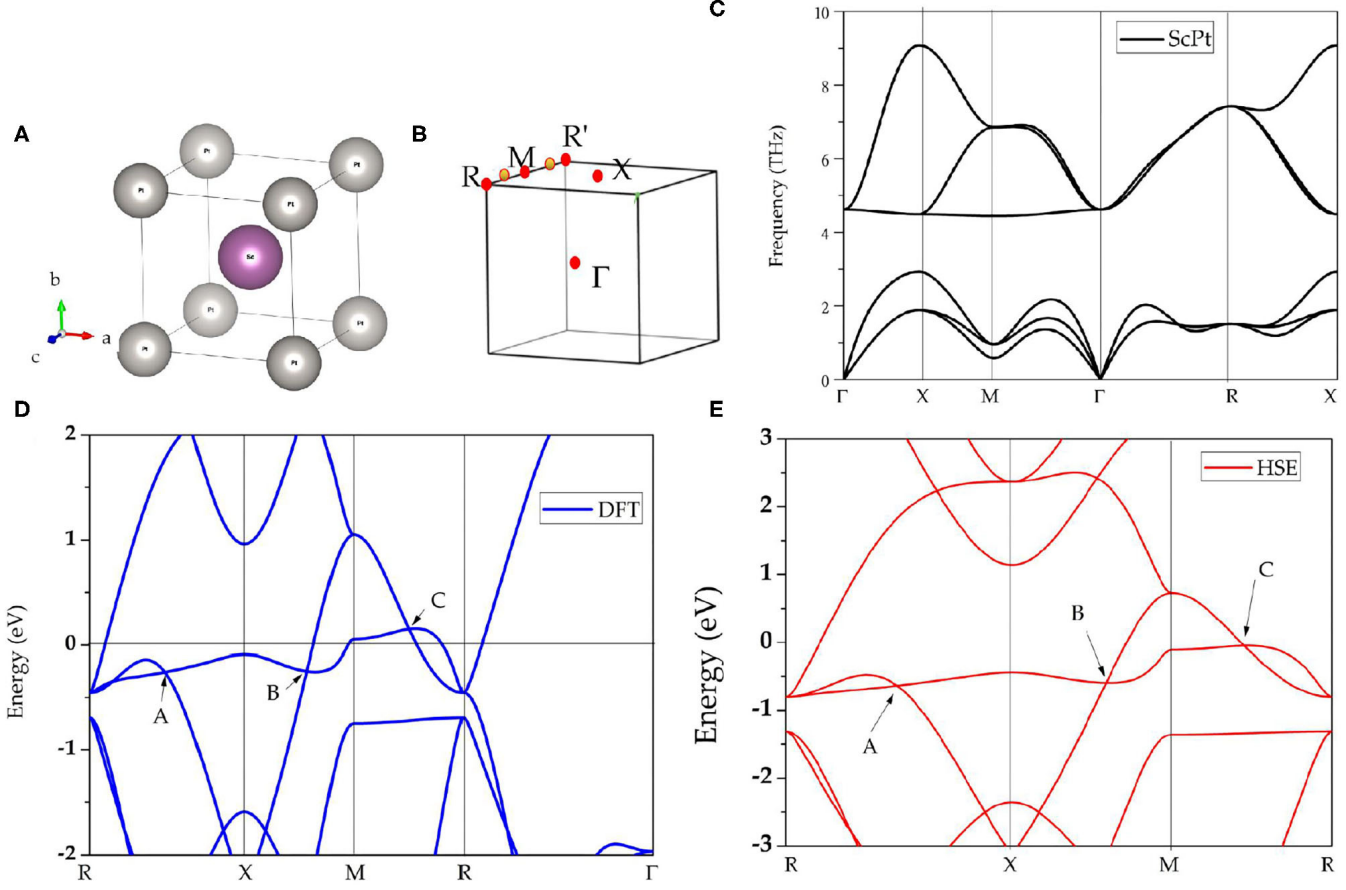
**(A)** Crystal structures of cubic ScPt; **(B)** Brillouin zone and the considered high-symmetry points Γ-X-M-Γ-R-X; **(C)** calculated phonon dispersion of cubic ScPt at its optimized lattice constant; **(D)** calculated band structure of cubic ScPt with PBE method, where **(A–C)** indicate the band-crossing points around the Fermi level; and **(E)** calculated band structure of ScPt with Heyd–Scuseria–Ernzerhof (HSE) screened hybrid functional.

The topological signatures of cubic-type ScPt, YPt, and LaPt are investigated. As an example, Figure 1A shows the structural model of cubic ScPt. This primitive ScPt cell contains one Sc atom and one Pt atom at the (0.5, 0.5, 0.5) and (0, 0, 0) sites, respectively. Using first-principles calculation, the structural models of ScPt, YPt, and LaPt are fully optimized; Table 1 lists the calculated results.

**Table 1:** Optimized lattice constants for ScPt, YPt, and LaPt.

**Compounds**	**a (Å)**	**b (Å)**	**c (Å)**
ScPt	3.283	3.283	3.283
YPt	3.488	3.488	3.488
LaPt	3.659	3.659	3.659

**Figure 2:**
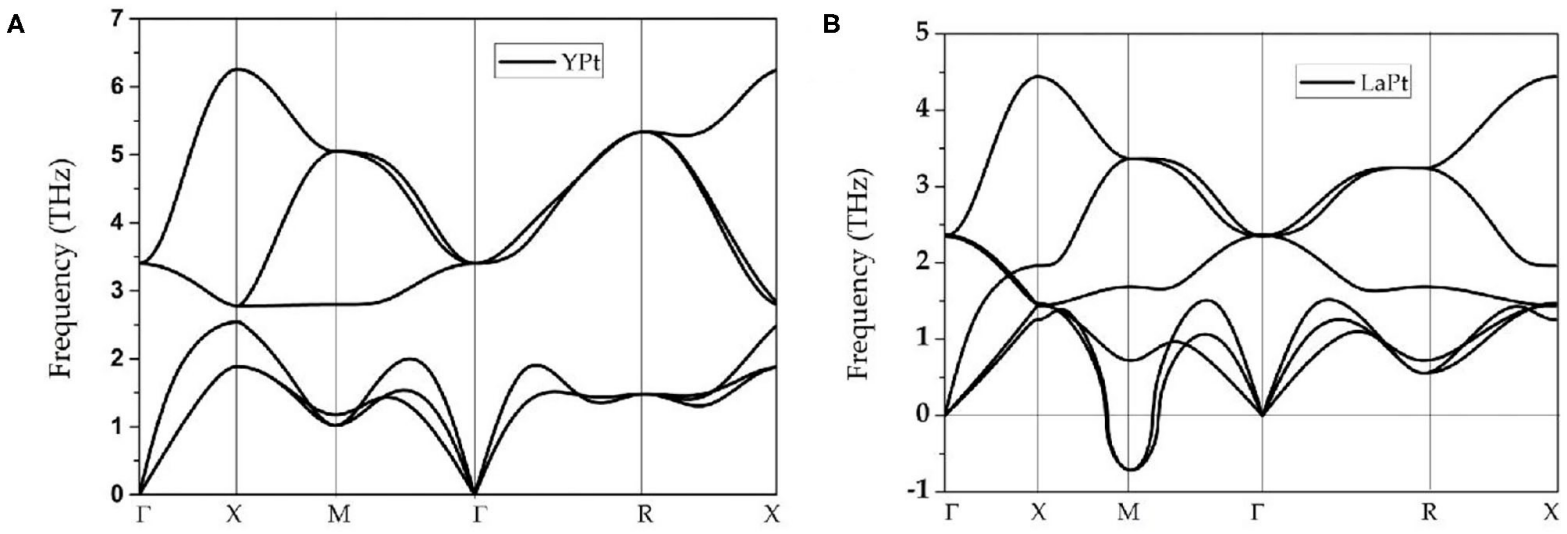
**(A,B)** Calculated phonon dispersion of cubic YPt and LaPt, respectively, at their optimized lattice constants. The phonon dispersions of both intermetallic compounds are obtained using the force-constants method with Phonopy code.

The phonon dispersions of cubic-type ScPt, YPt, and LaPt are calculated using the force-constants method with Phonopy code (Togo and Tanaka, 2015). For these three compounds, 2 × 2 × 2 supercells are built to calculate the phonon dispersions. The considered high-symmetry points are Γ-X-M-Γ-R-X, as shown in Figure 1B. Figures 1C, 2A exhibit the calculated phonon dispersions of ScPt and YPt; one can find that ScPt and YPt are dynamically stable owing to the absence of the imaginary frequency (Han et al., 2019; Wu et al., 2019). However, the obtained phonon dispersion shown in Figure 2B indicates that LaPt is not dynamically stable.

## Computational Methods

In this study, first-principles calculations are used, and the generalized gradient approximation (GGA) (Perdew et al., 1996) of the Perdew–Burke–Ernzerhof (PBE) (Perdew et al., 1998) functional is adopted for the exchange-correlation potential. In the calculations, the cutoff energy is set as 600 eV, and the Brillouin zone is sampled using a Monkhorst-Pack *k*-mesh with a size of 9 × 9 × 9. To ensure good convergence, the calculations continue until the energy deviation is <10^−6^ eV/atom. The atomic positions and lattice constants of the structures were totally relaxed until all the force components were smaller than 10^−3^ eV/Å.

## Results and Discussion

**Figure 3:**
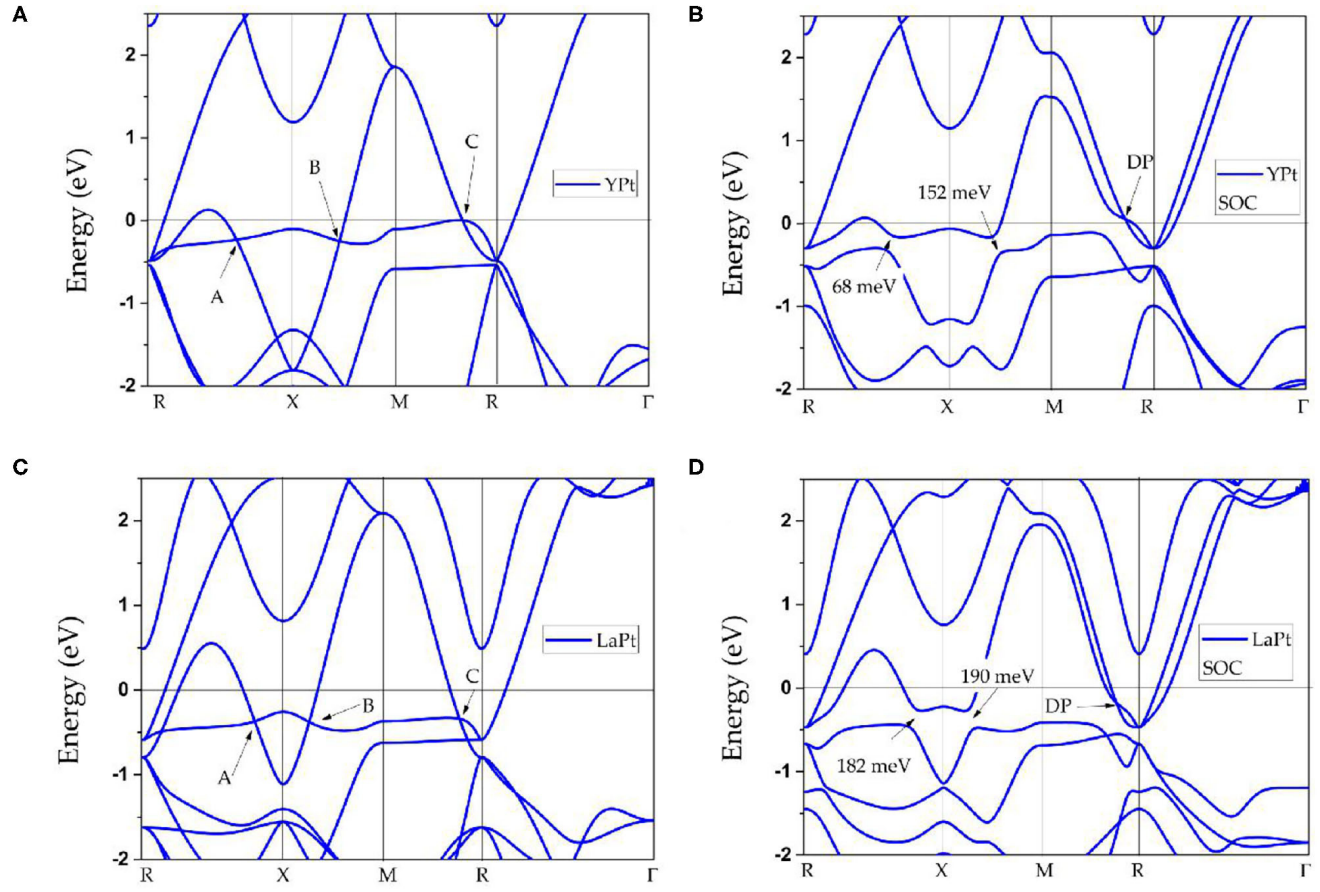
**(A–D)** Calculated band structures of cubic YPt and LaPt with their optimized lattice constants. The spin–orbit coupling effect is neglected for **(A,C)** and added for **(B,D)**.

First, the physical natures of ScPt, YPt, and LaPt are determined. Figures 1D, 3A,C, respectively, show the band structures of ScPt, YPt, and LaPt along the R-X-M-R-Γ paths. The spin–orbit coupling (SOC) is not added for the band structures in these figures; the effect of SOC on the electronic structures of these compounds will be discussed later in this paper. These three figures show that the bands and the Fermi level overlap each other, indicating common metallic behaviors.

Moreover, some obvious band crossings are seen around the Fermi level, namely, point A along the R-X path, point B along the X-M path, and point C along the M-R path. A careful study of these three band-crossing points indicates that points A and B are doubly degenerate band-crossing points, whereas point C is a 3-fold degenerate band-crossing point formed by a doubly degenerate band and a non-degenerate band. Apart from these three clear band-crossing points, the band structure near the Fermi level is very clean; therefore, these three points dominate the topological signatures of these compounds. For clarity, hereafter, ScPt is used as an example to investigate the band topology considering that the band structures of ScPt, YPt, and LaPt are almost the same near the Fermi level.

Figure 1E shows the band structure of ScPt with the revised Heyd–Scuseria–Ernzerhof (HSE) (Heyd and Scuseria, 2004) screened hybrid functional. The HSE method is well-known to be accurate for describing the band gap of topological materials. In particular, for some *d*-orbital-dominated systems, the GGA method cannot provide a fair evaluation of the band gap around the Fermi level. Figures 1D,E show that the band-crossing points A, B, and C are still maintained under the HSE method; this confirms that the GGA method is suitable for investigating the electronic structure of the ScPt system.

In addition to SOC, the ScPt system enjoys time reversal (*T*) and spatial inversion (*P*) symmetries. Basically, doubly degenerate band crossings like points A and B should not be isolated (Weng et al., 2016b; Zhang et al., 2018b). Instead, they should belong to one type of nodal structure; they most commonly belong to a nodal-line structure. As shown in Figures 1D, 3A,C, two bands cross each other and form two band-crossing points A and B along the R-X and X-M paths. Symmetry analysis shows that these two bands belong to irreducible representations *A*_1*g*_ and *A*_2*u*_ of *D*_4*h*_ symmetry, respectively.

**Figure 4:**
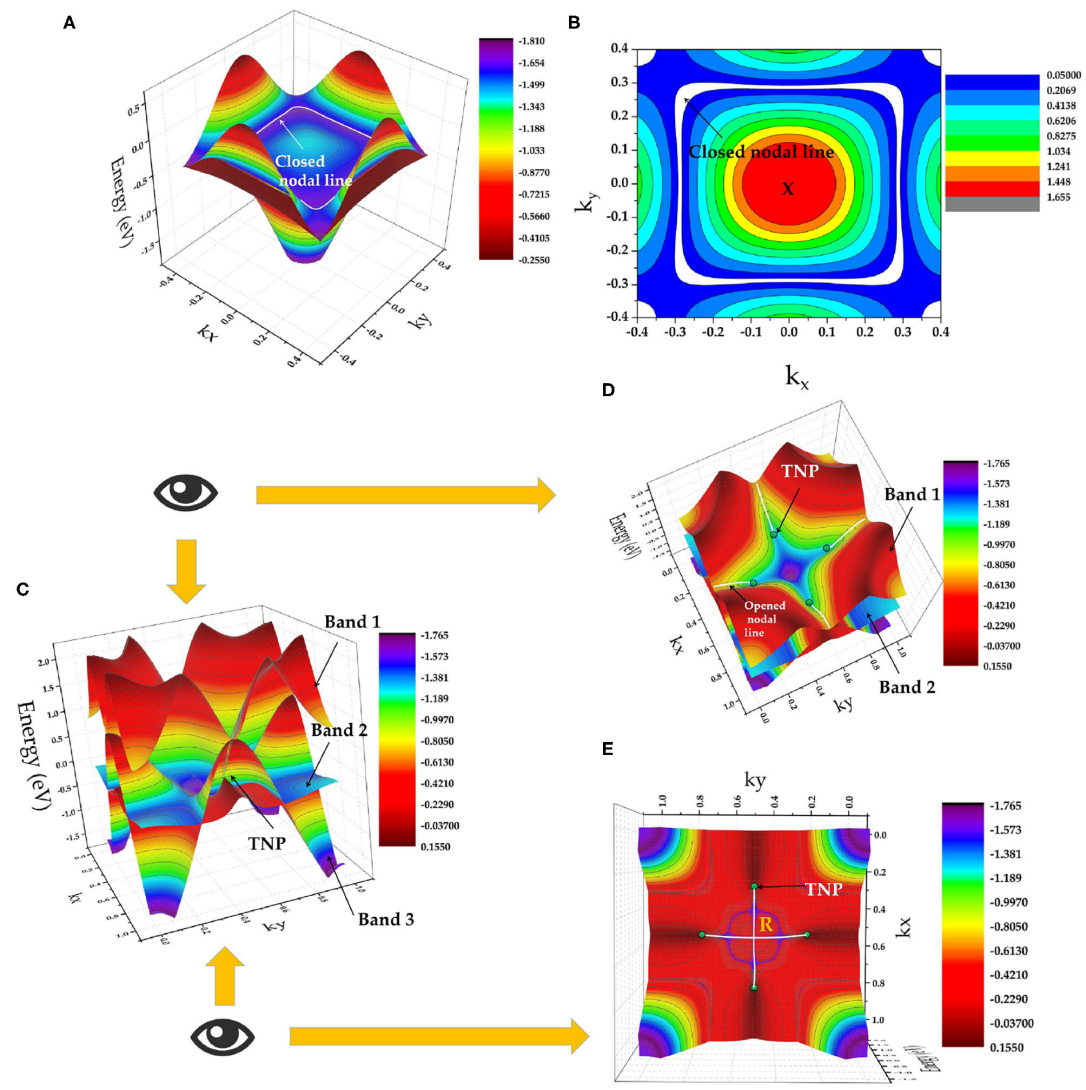
**(A)** X-centered 3D band dispersion in the k_z_ = π plane; **(B)** shape of closed X-nodal line in the k_z_ = π plane (nodal line is indicated by white lines and marked by arrows); **(C)** R-centered 3D band dispersion in the k_z_ = π plane, where TNPs and bands 1, 2, and 3 are marked by arrows; **(D)** top view of **(C)**; and **(E)** bottom view of **(C)**. The TNPs and the opened nodal lines (formed by degenerate bands 1 and 2) are indicated by green balls and white lines, respectively.

Figure 4A shows the X-centered 3D band dispersion of the k_z_ = π plane. The two above-mentioned bands form a closed nodal line in the k_z_ = π plane (highlighted by a white line), and points A and B belong to this closed nodal line. The crystal symmetry of the ScPt cubic system implies three closed nodal lines in the k_x/y/z_ = π planes. The nodal lines are located in the mirror-invariant plane, and they protect the mirror symmetry M_x,y,z_. As an example, Figure 4B shows the shape of the X-centered closed nodal line in the k_z_ = π plane.

Figure 4C shows the R-centered 3D band dispersion in the k_z_ = π plane; here, TNPs are indicated by green balls. As shown in Figures 1D, 3A,C, the band-crossing point C is formed by a 2-fold degenerate band and a non-degenerate band along the M-R path. This 2-fold degenerate band can be seen as two independent bands that are completely degenerated along the whole M-R path. Therefore, this 2-fold degenerate band (named as band 1 and band 2) should contain a series of band-crossing points along the whole M-R path and form an opened nodal line along the M-R path. To clearly present the opened nodal lines and the TNPs in ScPt, Figures 4D,E, respectively, show top and bottom views of Figure 4C. These figures clearly show the opened nodal lines formed from bands 1 and 2 along the R-M path as well as the TNPs. Therefore, ScPt, YPt, and LaPt are topological metals that co-exhibit opened and closed nodal lines when the spin-orbit coupling is ignored. Based on the above-mentioned results, the ScPt family of materials is a good platform to study the relationship of closed and opened nodal lines.

Moreover, band-crossing point C along the M-R path is a TNP. Normally, TNP can not only occur in isolation but also be linked by nodal lines in the momentum space. One pair of TNPs exists in ScPt, YPt, and LaPt. Figure 1B shows a schematic of the pair of TNPs (indicated by yellow balls) along the R-M-R′ path. As shown in Figure 4C, the TNPs are located at the crossing of band 3 (non-degenerate band) and bands 1 and 2 (2-fold degenerate band). Therefore, ScPt is concluded to have one-dimensional topological elements, namely, opened and closed nodal lines (in the k_x/y/z_ = π plane) and a zero-dimensional topological element, namely, TNP, along the R-M-R′ path when the spin-orbit coupling is ignored. Therefore, ScPt, LaPt, and YPt are excellent target materials for studying the entanglement between nodal-line and nodal-point fermions.

**Figure 5:**
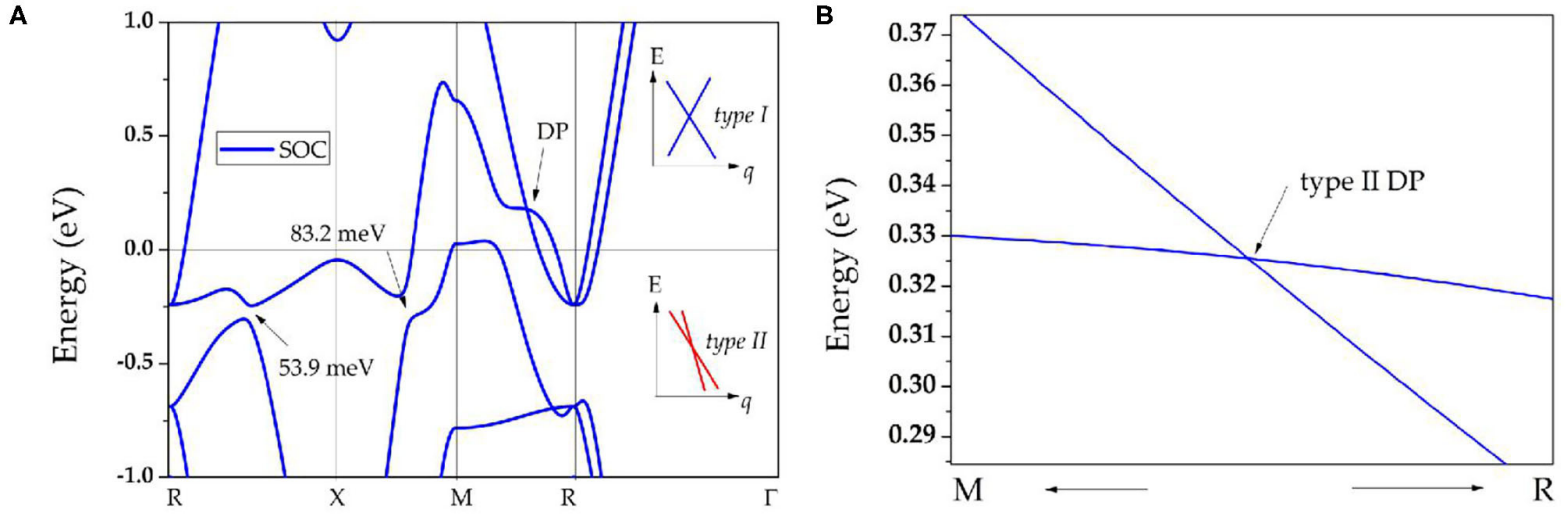
**(A)** Band structure of ScPt with spin–orbit coupling; the insets show schematics of types I and II nodal points, indicated by blue and red lines, respectively; for type I nodal points, they are conventional type band dispersion, however, for type II nodal points, they enjoy a tilted band dispersion; **(B)** enlarged band structure of ScPt along the M-R path slightly above the Fermi level.

Finally, the effect of SOC on the band structures of ScPt, YPt, and LaPt is investigated. The corresponding results are shown in Figures 5A, 3B,D, respectively. The gaps induced by SOC for band-crossing points A and B are 53.9 and 83.2 meV, 68 and 152 meV, and 182 and 190 meV for ScPt, YPt, and LaPt, respectively. In comparison, the gaps induced by SOC in the well-known nodal-line materials Cu_2_NPd (Yu et al., 2015), CaAgBi (Chen et al., 2017b), and BaSn_2_ (Huang et al., 2016) are 60–100 meV, 80–140 meV, and 60–160 meV, respectively. Therefore, ScPt and YPt are comparable to these reference materials.

Moreover, Figures 5B, 3B,D show that the TNPs disappear in ScPt, YPt, and LaPt systems. However, a new topological signature reveals a nodal point with linear band dispersion around the Fermi level. When the SOC effect was considered, each band was doubly degenerate. Therefore, the newly occurring nodal point along the along R-M path should be a Dirac nodal point (DP) with 4-fold degeneracy. Specifically, a pair of DPs with 4-fold degeneracy is found along the R-M-R′ path. Notably, similar SOC-induced TNP–DP transitions have also been reported in ErAs (Meng et al., 2020a), TiB_2_ (Zhang et al., 2017d), and Li_2_NaN (Jin et al., 2019b) topological materials. However, unlike the type-I DP predicted for ErAs, this is a type-II DP that may show strong anisotropy (Zhang et al., 2018b).

## Summary

In summary, cubic-type ScPt, YPt, and LaPt are shown to be newly designed topological materials through the use of density functional theory. ScPt and YPt are dynamically stable whereas LaPt is not. Without SOC, XPt (X = Sc, Y, La) metals show closed and opened nodal-line states and one pair of TNPs. With SOC, the TNPs (along the R-M-R′ path) change to type-II DPs and the nodal-line states in k_x/y/z_ = π planes are gapped. A series of interesting topological signatures has been predicted in XPt (X = Sc, Y, La), and it is hoped that these proposed topological elements can be confirmed through experiments in the future.

## Data Availability Statement

The original contributions presented in the study are included in the article/supplementary materials, further inquiries can be directed to the corresponding author/s.

## Author Contributions

The author confirms being the sole contributor of this work and has approved it for publication.

## Conflict of Interest

The author declares that the research was conducted in the absence of any commercial or financial relationships that could be construed as a potential conflict of interest. The handling editor declared a past co-authorship with one of the authors, HX.
